# Managing Implementation of a Parental Support Programme for Obesity Prevention in the School Context: The Importance of Creating Commitment in an Overburdened Work Situation, a Qualitative Study

**DOI:** 10.1007/s10935-020-00584-2

**Published:** 2020-03-10

**Authors:** Helena Bergström, Elinor Sundblom, Liselotte Schäfer Elinder, Åsa Norman, Gisela Nyberg

**Affiliations:** 1 Department of Global Public Health, Karolinska Institutet, Tomtebodavägen 18A, 171 77 Stockholm, Sweden; 2 Academic Primary Health Care Centre, Region Stockholm, Solnavägen 1E, 113 65 Stockholm, Sweden; 3 Centre for Epidemiology and Community Medicine, Region Stockholm, Solnavägen 1E, 113 65 Stockholm, Sweden; 4 The Swedish School of Sport and Health Sciences, Lidingövägen 1, 114 33 Stockholm, Sweden

**Keywords:** Barriers, Diet, Facilitators, Physical activity, Qualitative, School children

## Abstract

Health-related behaviours in children can be influenced by parental support programmes. The aim of this study was to explore barriers to and facilitators for the implementation of a parental support programme to promote physical activity and healthy dietary habits in a school context. We explored the views and experiences of 17 coordinating school nurses, non-coordinating school nurses, and school principals. We based the interview guide on the Consolidated Framework for Implementation Research. We held four focus group discussions with coordinating and non-coordinating school nurses, and conducted three individual interviews with school principals. We analysed data inductively using qualitative content analysis. We identified “Creating commitment in an overburdened work situation” as an overarching theme, emphasising the high workload in schools and the importance of creating commitment, by giving support to and including staff in the implementation process. We also identified barriers to and facilitators of implementation within four categories: (1) community and organisational factors, (2) a matter of priority, (3) implementation support, and (4) implementation process. When implementing a parental support programme to promote physical activity and healthy dietary habits for 5- to 7-year-old children in the school context, it is important to create commitment among school staff and school nurses. The implementation can be facilitated by political support and additional funding, external guidance, use of pre-existing resources, integration of the programme into school routines, a clearly structured manual, and appointment of a multidisciplinary team. The results of this study should provide useful guidance for the implementation of similar health promotion interventions in the school context.

## Background

Because health-related behaviours and obesity track from childhood to adolescence and adulthood, it is highly important to promote and establish healthy eating habits and physical activity among children (Biddle, Pearson, Ross, & Braithwaite, 2010; Craigie, Lake, Kelly, Adamson, & Mathers, 2011; Singh, Mulder, Twisk, van Mechelen, & Chinapaw, 2008; Telama, 2009). The health of children in Sweden is relatively good, as compared to other countries, but there is a steep social gradient in the prevalence of overweight and obesity among children from families with low socioeconomic status (SES; Elinder, Heinemans, Zeebari, & Patterson, 2014; Magnusson, Hulthen, & Kjellgren, 2005; Moraeus et al., 2012; Sundblom, Petzold, Rasmussen, Callmer, & Lissner, 2008). Low parental education has been associated with a higher intake of unhealthy foods and a lower intake of vegetables among children (Safsten, Nyberg, Elinder, Norman, & Patterson, 2016), as well as with less participation in organised sports and more time spent watching TV (Hesketh, Crawford, & Salmon, 2006; Moraeus et al., 2012). According to a study of health behaviours among Swedish school children, those whose parents had attained higher levels of education had more favourable health-related behaviours than those with less educated parents in seven out of 12 health behaviours related to diet and physical activity (Elinder et al., 2014). It is therefore important to develop and implement health interventions that are effective among disadvantaged groups, and do not widen the socioeconomic health gap.

The World Health Organization has released an action plan called “Ending childhood obesity” that includes six main recommendations, in which the role of parents, families, caregivers, and educators in encouraging healthy behaviours among children is strongly emphasised (World Health Organization, 2016). Parents can make healthy foods and activities accessible to their children (Ferreira et al., 2007), and employ parenting styles and practices to support and encourage healthy habits (Collins, Duncanson, & Burrows, 2014; Davison, Cutting, & Birch, 2003; Seabra et al., 2013; Ventura & Birch, 2008; Vollmer & Mobley, 2013). A majority of obesity prevention interventions in children are school-based (Lobstein et al., 2015). Evidence has accumulated that health promotion in schools can enhance children’s physical activity and healthy dietary habits, although the effects achieved are usually small and short-lived (Brown & Summerbell, 2009; Dobbins, Husson, DeCorby, & LaRocca, 2013; Peirson et al., 2015; Waters et al., 2011) and should therefore be complemented by program components that target parents. A systematic review of parental support programmes has showed that merely sending home information is ineffective, whereas parental counselling, either face-to-face or by telephone, is effective in changing children’s diet but not physical activity (Kader, Sundblom, & Elinder, 2015). Some weak effects on body mass index (BMI) have been obtained by group-based interventions (Kader et al., 2015). Furthermore, effectiveness was generally higher in studies targeting parents of preschool age children (2–5 years) than those targeting parents of older children.

The parental support programme “A Healthy School Start” (HSS) was developed and evaluated with the aim of promoting physical activity and healthy dietary habits and prevent obesity, especially among children in disadvantaged areas where the prevalence of overweight and obesity is high (Nyberg et al., 2015; Nyberg, Norman, Sundblom, Zeebari, & Elinder, 2016). This universal programme, which targets 6 year olds and their parents regardless of the weight status of their children, is described in detail elsewhere (Nyberg, Sundblom, Norman, & Elinder, 2011). Briefly, the programme comprises three components: (1) a brochure containing health information for the parents, (2) two individual sessions of Motivational Interviewing (MI) for the parents, and (3) ten 30-min teacher-led classroom activities for the children. All three components target the parents: the first two directly and the third indirectly through the children’s homework. MI is a client-centred and goal-steering method to support an individual in behaviour change ( Miller & Rollnick, 2013). During the first MI session the parents choose some aspect of their child’s diet, physical activity, or sleep that they want to change, and during the second session they explore their efforts towards achieving this goal. The MI sessions should preferably be conducted by MI-trained staff. For classroom activities, the teachers are provided with a manual and a tool-box with pedagogic materials regarding diet, physical activity, and sleep, and the children receive homework assignments to carry out together with their parents at home.

The HSS programme has been evaluated within the context of two cluster randomised controlled trials (Nyberg et al., 2015, 2016). The mean age of the children in the first trial was in 6.2 years and 6.3 years in the second trial. Outcomes were in both trials measured before and after intervention, and at follow-up after 5 months. Results from the first study, carried out in an area with low to medium SES in Stockholm, Sweden, showed positive intervention effects on vegetable intake and physical activity among girls during weekends (Nyberg et al., 2015).

Results from the second study, carried out in an area with low SES and a high prevalence of overweight and obesity, showed positive intervention effects, e.g., lower consumption of unhealthy foods and unhealthy drinks, as well as lower BMI z-scores, among children who were obese at baseline (Nyberg et al., 2016). No effect could be seen on physical activity for the whole group in either of the studies. This was probably due to the fact that the majority of the children in both studies had already reached the recommendations for physical activity at baseline. However, as physical activity levels among children peak at the age of 5–6 and decrease rapidly thereafter (Cooper et al., 2015), it is still important to promote physical activity from an early age. The positive effect of vegetable intake was sustained among boys at 5 months follow-up in the first study, and the beneficial effect on the consumption of unhealthy food was sustained among boys at 5 months follow-up in the second study, while other effects tended to wear off. Thus, the results of the two trials are in agreement with the international literature, according to which diet—and to a lesser extent BMI—can be positively influenced through parental counselling (Kader et al., 2015), while physical activity is more difficult to influence at this age. However, the effects of the programme levelled off after 5 months, and we have therefore reasoned that the HSS programme may have to be extended and/or intensified if we are to enhance and sustain its effectiveness.

In both trials, the programme was conducted with substantial support from the research team, and the MI sessions were conducted by MI-trained external staff (Nyberg et al., 2015, 2016). In order for this programme to be implemented on a broader scale it has to be fully integrated into the school context, and the most realistic solution is that school nurses should provide the parental counselling, which means that they have to be competent in MI. Health promotion is emphasised in the Swedish guidance to school health services (The National Board of Health and Welfare & The Swedish National Agency for Education, 2016). School guidelines state that “student health care should mainly work with health promotion and prevention” and that the schools have the potential to support healthy diet and physical activity, for example by “giving parental support that encourages healthy dietary habits, physical activity and less sedentary behaviour” (pp. 27 and 98).

No matter how effective a programme might be, it will only result in health changes at a population level if widely and well implemented. The aim of this study was to explore barriers to and facilitators for the implementation of a parental support programme aimed at promoting physical activity and healthy dietary habits in the school context and carried out by school staff, as perceived by school nurses and school principals. The HSS programme was used as an example of such a programme.

## Methods

### Study Design

We used an inductive qualitative design to explore the views and experiences of school nurses and school principals. Qualitative methods are useful for exploring perceived barriers to and facilitators of implementation (Landsverk et al., 2012) and they permit the researcher to study selected issues in depth (Patton, 2015).

### Setting and Participants

Student health care in Sweden comprises medical, psychological and psychosocial actions as well as special education (The National Board of Health and Welfare & The Swedish National Agency for Education, 2016). School nurses and school doctors cooperate with psychologists, counsellors and all other professional groups in school, often organised into a school health care team. The professionals within the team are either employed directly by the schools or are brought in from providers within the municipality. Each municipality in Sweden has a coordinating school nurse, who coordinates the work of the non-coordinating school nurses in that municipality. The number of students per school nurse varies widely. Health promotion is included in the national guidelines of the school health care, but mandatory tasks such as health checks (height, weight and eye tests) and vaccination are always prioritized.

To obtain different perspectives, and achieve triangulation to support trustworthiness (Patton, 2015), we collected data from coordinating school nurses, non-coordinating school nurses, and school principals. None of these had previously participated in the HSS intervention. The reason for choosing participants who had not taken part in the HSS intervention previously was to obtain a broad perspective on implementation from school principals and school nurses working in different municipalities. The coordinating school nurse in each of the 26 municipalities in Stockholm County was contacted by email and invited to participate in the study. Those who expressed interest were then asked to contact school nurses and school principals within their municipality. We used convenience sampling, as the school nurses and school principals have a heavy workload and are thus difficult to reach for interviews. To assure a wide range of views, we asked the coordinating school nurses to invite school nurses and school principals from areas with varying SES within their respective municipalities.

Our study collected data from 17 informants: four coordinating school nurses, ten school nurses and three school principals. Ten of the 14 school nurses had some previous training in MI. The length of their MI training ranged from a few lectures as part of their basic education to 3 days plus follow-up. All participants but one stated that MI was already used in their school context. The nurses represented seven different municipalities from local areas varying in SES. The school principals represented schools in areas with medium to high SES, covering three different municipalities. According to national statistics the 13 schools represented by school nurses and school principals had between 280 and 700 students in year 1 through 10 (children aged 6–15 years). The 13 schools had a wide range (8–85%) in the proportion of foreign-born students or foreign-born parents. Students with parents who had completed post-secondary education ranged from 48 to 89% across schools. (SIRIS, 2016). None of the participants had taken part in or had any prior knowledge of the HSS programme. A week before the interview or focus group, participants received a copy of the HSS manual. At the time of the interview or group discussion the participants were given a brief presentation to the programme and the three components.

### Data Collection

In order to capture the views and experiences of school nurses, we chose a focus group methodology. In focus groups, the interaction between the group members is highlighted and it is therefore a suitable method to explore motivation, behaviour, and attitudes in a group (Krueger & Casey, 2014). We conducted one focus group with the coordinating school nurses and three focus groups with the non-coordinating school nurses, because homogeneous groups in terms of occupation and education are generally recommended (Krueger & Casey, 2014). Our aim was to recruit four to six participants for each group, as this group size is comfortable for the participants (Krueger & Casey, 2014), but in one case, the group ended up with fewer respondents (*n* = 2) due to difficulties in finding a suitable date and place for the participants. We included between two and four participants in each focus group discussion, which lasted between 75 and 100 min. We conducted the discussions in places suggested by the participants, mainly in schools. These groups were led by a moderator (first author HB) and an assistant moderator (second author ES or other colleague). We interviewed school principals individually, as this is a useful method when the purpose is to access people’s personal perspectives (Patton, 2015). The interviews lasted from 45 to 60 min and were conducted in a place chosen by the respondent, usually an office or a meeting room at his/her school.

We developed two different semi-structured interview guides, one for the coordinating school nurses and the non-coordinating school nurses and another one for the school principals. The questions in the interview guides were concerned with the implementation of the parental support program to support healthy diet and physical activity, such as the HSS programme, and were based on the five domains included in the Consolidated Framework for Implementation Research (CFIR), as these domains are known to affect implementation of interventions (Damschroder et al., 2009). The five domains are: (1) intervention characteristics (e.g., the content, format, quality and structure of the intervention itself); (2) the outer setting (e.g., the economic, political and social context in which the intervention is delivered); (3) inner setting (e.g., the structural and cultural contexts within the organisation delivering the intervention); (4) the characteristics of individuals (e.g., deliverers’ norms, skills, and interests); and (5) the implementation process (e.g., planning, engaging, executing and evaluating the intervention). The interview guides are available from the corresponding author upon request. One of the authors (HB) facilitated all focus groups and interviews were conducted between January and March 2016. Both focus group discussions and individual interviews were audiotaped and transcribed by HB.

### Data Analyses

We read through the transcripts several times to obtain a sense of the content. Then we analysed them manually, without any software. Initially, we analysed the transcripts separately for coordinating school nurses, non-coordinating school nurses and school principals to detect possible differences among the three groups. As we found none, we then integrated the analyses into a coherent whole, but retained the initial separate analyses to facilitate a description of minor differences among the groups. We analysed data inductively using qualitative content analysis according to the procedure described by Graneheim and Lundman (2004). First, we identified meaning units and labelled them with codes. We compared the codes based on differences and similarities and sorted them into categories and subcategories. Finally, we identified a theme, based on the content of the categories. We defined the theme, as well as categories and sub-categories, by intersubjective agreement among the authors, to enhance the trustworthiness of the study (Patton, 2015). In cases of disagreement, we carefully reread the transcripts until consensus on the categorisation was reached. Data from focus group discussions comprised interactions between participants. Hence, our analysis of group discussions focused on the interaction within the groups (Krueger & Casey, 2014), and we chose quotes from these group interactions rather than from individual statements.

## Results

**Fig. 1:**
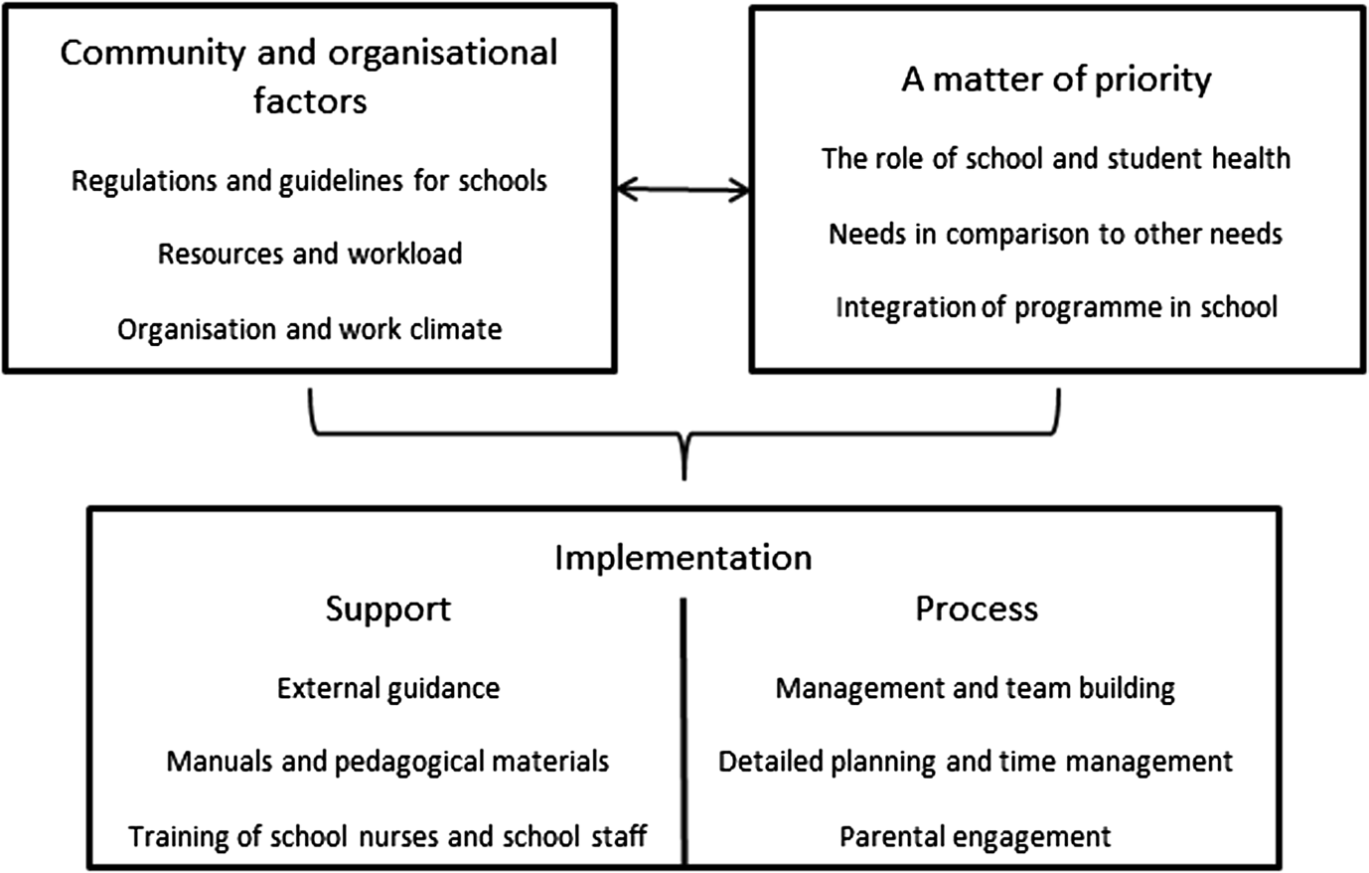
Descriptive categories of barriers and facilitators for the implementation of a parental support programme

We identified an overarching theme at a latent level—Creating commitment in an overburdened work situation—and four descriptive categories at a manifest level: (1) community and organisational factors, (2) a matter of priority, (3) implementation support, and (4) implementation process. An overview of the results is given in Fig. 1. The hierarchy of the figure describes how community and organisational factors, as well as priorities, influence opportunities for implementation. Barriers and facilitators within each category are presented below.

### Creating Commitment in an Overburdened Work Situation

A successful implementation of a parental support programme to promote physical activity and healthy dietary habits in school constitutes a challenge because of an overburdened work situation for school staff and school nurses. Resources are scarce and the workload is heavy and many different tasks and health needs compete for attention. When implementing a programme that is not mandatory, it is important to create commitment among all staff members at the school, by giving them support and including them in the implementation process.

### Community and Organisational Factors

The possibilities for implementation of a parental support programme were described by the respondents as being governed by regulations and guidelines for schools, resources, and staff workload, as well as organisational and work climate. The potential barriers described mainly involved financial and time constraints and a heavy workload. *“*If we had twice as much school nurse time, then I think it would work great. Then I think we could also work more proactively with health promotion in general” (School principal 3).

The conditions described differed widely from school to school. In some schools, the staff experienced exhaustion related to organisational changes, cooperation difficulties between professions, or challenges related to administration and scheduling. A top–down decision to conduct the programme could be seen as both a barrier and a facilitator. The school principals perceived that they had the power to implement the program both with and without local policy support, and expressed a fear that a top–down decision would lead to less acceptance among staff compared to a bottom–up initiative. Conversely, school nurses perceived that a political decision would facilitate implementation as it would otherwise depend heavily on the school principals. The financial support that would be connected to a political decision was perceived as an important facilitator by all.

To some extent, it might be good if there’s like a decree that the schools have to implement it. Otherwise it’s all up to the school principals and their interests, what they think is important and fun. And maybe the [school’s] economy too, because that’s so different. If it’s supposed to be the same for everyone, it’s probably important to have a financial decision. (Focus group 2, School nurses)

Within the organisation, several facilitators could be identified, such as competent and engaged staff, the multidisciplinary school health team, supportive school principals, municipality networks for school nurses and school principals, and web-based systems for documentation and communication, as well as experts within the municipalities, such as public health planners or health educators.

### A Matter of Priority

A school’s choice of whether or not it will implement a parental support programme to improve diet and physical activity depends on how it views its own role and that of school health care, how it rates the needs regarding diet and physical activity compared to other health needs, and how it views the possibilities of integrating the programme into the school context. Barriers include hesitation about whether parental support is actually a prioritised task for the school. It was argued that schools must focus on their main task: educating students.

Yeah, I think about what the school’s role is. I sometimes feel, like, are we supposed to be responsible for educating the parents as well? We want to focus on our students, and obviously working in partnership with the parents benefits the students. For example, if we’re working on reading, then obviously if they read at home then that’s great. I’m also thinking about, like where to draw the line, there’s a lot of stuff that the parents want us to teach them. (School principal 1)

It was also argued that there are other needs to consider, such as poor mental health, problems related to immigration, and poor school performance. If there is a risk that the programme will end up being a short-lived project this is also a barrier, because schools only want to invest their scarce resources in long-lived programmes that can be integrated into school routines.

Perceived facilitators included the fact that health promotion is already a natural part of everyday work for schools and student health care. There was a shared view that good health contributes to the achievement of learning goals. Overweight and obesity were perceived as an alarming health problem among students, as were inactivity and unhealthy eating habits. School nurses and school principals expressed concerns about children eating meals in solitude, eating selectively and spending excessive amounts of time in front of TV/computer screens, and therefore felt motivated to engage in a programme targeting these issues. Poor mental health was viewed as one of the most pressing health problems among students, but respondents also saw mental health as being intertwined with diet and physical activity and felt that these health issues interact.

1:1 Also, I think if you think about the stress in society, it’s really closely connected with what food you serve. It used to be, you could be making dinner for three hours, and now you only spend fifteen minutes or something.

1:2 You don’t even have time to boil potatoes.

1:1 So it’s hard to separate, everything is linked together.

1:3 We can see it’s all connected, but I don’t see that when I read articles or look at the latest literature. There you don’t see the big picture. It is not as if we get something that says “and if you eat better, you will also feel better mentally.” I mean, it’s more like we’re the ones that understand. But that’s not how it’s presented.

1:4 But it is connected. (Focus group 1, Coordinating school nurses)

### Implementation Support

To be able to implement a parental support programme within schools, respondents expressed a need for support, in terms of guidance, manuals, and pedagogical materials as well as training for school nurses and school staff. Possible barriers identified included the cost of MI training for school nurses and the risk that staff members will view manuals and materials as an extra burden. One suggestion was that the information brochure should not be sent to parents but handed over in person, otherwise it might be thrown away or forgotten. Finally, respondents suggested that kick-off meetings should be arranged by someone from outside the school, as it was viewed as a potentially challenging task for the principal or the school nurse to get all school staff committed to the programme.

Sometimes I feel like a broken record on teacher training days. So there you are again, trying to look thrilled and present something totally new, and it feels like, oh God do I have to dress up like a clown and try to launch something again*?* (School principal 2)

Participants expressed a need for the components of the programme to be standardised and clear. Implementation was believed to be facilitated by a programme with a clear structure and a manual including checklists, time plan, costs, questions to ask during MI sessions with parents, and support for evaluation.

3:1 Actually, it should be more similar, so you don’t do it in different ways, or have a possibility of doing it different ways. So it’s more like “this is how we do it and when we do it,” and so on. As I see it, with that little experience I have of working as a school nurse, everyone is in their own little universe, arranging their work on their own. You know more or less what you’re supposed to do, what your mission is… I think it’s more that it shouldn’t be possible to do things very differently, that you have a time frame and how it should be structured. Yeah, a structure.

3:2 Oh, I agree with you there, absolutely. Crystal clear. It should be crystal clear. So, like sometimes you can feel like, oh God, that’s ridiculous that this is supposed to take 15 minutes. But, yes, I think it’s really important. (Focus group 3, School nurses)

Prior to implementation, school nurses would need basic or follow-up training in MI and teachers would need training sessions to learn about and discuss the classroom component. According to school nurses and school principals, implementation would also be facilitated by a contact person who can be reached by telephone or email, when needed. Finally, the school nurses wished for additional information materials to use when presenting the programme to parents, such as ready-to-use presentations, a poster and/or a movie.

### Implementation Process

The respondents conceptualized several parts of the implementation process; including management and team building, detailed planning and time management, and parental engagement. Barriers described included the risk that the school nurse could be left alone with the responsibility for the programme, and difficulties arranging two MI sessions with the parents, because the work situation of school nurses is strained. Reaching and engaging the parents was also viewed as a challenge; because parents often do not show up at school information meetings. Barriers described in the context of contact with parents included lack of interest, ignorance, language barriers, lack of time, social problems, and difficulties discussing the topic, as it can be viewed as sensitive.

4:1 If there’s ever a time, then surely it would be at school information meetings you might be able to reach everyone, at least everyone who shows up. But otherwise, like one night in school when parents were supposed to discuss values or something, it drew a handful of parents from the entire school. An issue you would think would be really important.

4:2 Yeah, it’s a pity, because I think the only way to get something done is to talk to the parents. You can’t talk to the kids yet for many years, and they don’t decide what to bring home from the shops or which rules should be applied.

4:3 Then it’s also difficult with the language. This a multicultural area and it’s difficult with the language. They don’t know the language and need interpreters and so on. It’s difficult then, I think. (Focus group 4, School nurses)

The respondents believed that the implementation could be facilitated by a multidisciplinary team consisting of a person who has the main responsibility for the programme, a supportive principal, and networks within the municipality where school nurses, school principals and teachers can discuss and exchange experiences with their colleagues from other schools. The implementation process was also expected to be facilitated by commitment among local decision-makers and school management, engagement of other school staff and a detailed time plan. As it was not always regarded as feasible to have two MI sessions with all parents, one suggestion was to offer the initial MI session to all parents and the follow-up session only to parents with an identified need. It was emphasised that it is important to get all staff committed, to offer them possibilities to discuss the implementation of the programme, and to plan for follow-up and evaluation.

It is a prerequisite that I perceive a need. The other option is “You must do this at school.” But if I have realised that there is a need, then it’s all about creating an understanding of what we should do. In other words, call a meeting and talk with those who are affected. And have an open discussion about how we can implement it. Like, here’s the problem, how shall we implement this? (School principal 3)

## Discussion

In this study, we explored barriers to and facilitators for the future implementation of a parental support programme in school addressing diet and physical activity, as perceived by school nurses and school principals. The overarching theme that emerged was that it is important to create commitment among all staff members in school and student health care to successfully implement a parental support programme in a context where the workload is generally high. We identified four categories at a manifest level, which we have also attempted to refer back to CFIR (see Table 1). This is recommended by the developers of CFIR in order to promote the ability to compare research over time and across contexts (Kirk et al., 2016).

**Table 1:** Correspondence between results from inductive analysis and consolidated framework for implementation research (CFIR) domains and constructs

Categories identified in inductive analysis	CFIR domain	Corresponding CFIR constructs
Community and organisational factors	Inner setting	Structural characteristics
		Network and communication
		Culture
		Implementation climate
		Readiness for implementation
		Leadership engagement
		Available resources
	Outer setting	External policy and incentives
	Characteristics of individuals	Other personal attributes (such as motivation and competence)
A matter of priority	Inner setting	Implementation climate
		Tension for change
		Compatibility
		Relative priority
	Outer setting	Patients’ needs and resources
Implementation support	Intervention characteristics	Complexity
		Design, quality and packaging
		Cost
	Process	External change agent
Implementation process	Process	Engaging
		Formally appointed internal implementation leaders
		External change agents
		Reflecting and evaluating

Both school nurses and school principals reported that their schools’ resources are limited, and that the workload is heavy, which in CFIR terms relates to the domain ‘Inner setting’ of the schools. This was also stressed by teachers in a previous implementation study of the HSS programme (Bergstrom et al., 2015). Limited resources for school health professionals have also been described in a study from the United Kingdom, where lack of capacity, among other things, constituted a barrier for health promotion activities (Turner et al., 2016). In this overburdened work situation, it is a challenge for the staff to engage in and commit to a programme that requires time and effort. Therefore, to be able to implement a programme like HSS with high fidelity, additional resources would be needed. On the positive side, the existing organisation offers great opportunities, such as engagement, competence, and structures for cooperation and communication between different professions both within and among schools, all of which could facilitate commitment among the staff. High quality formal communication and peer collaboration are known to contribute to effective implementation in general (Damschroder et al., 2009). Multidisciplinary collaborative approaches and professional networks have also been highlighted as one of five enablers regarding implementation of the health-promoting school concept (Hung, Chiang, Dawson, & Lee, 2014). Networks and pre-existing teams might form a solid basis for shared decision-making, which is important not only for a successful implementation but also for program sustainability (Durlak & DuPre, 2008).

Schools are continually offered various kinds of programmes and must prioritise, due to limited resources and a heavy workload. To implement a programme like HSS, schools must recognise a need, which in this case is closely connected to the prevalence of overweight and obesity among the students (CFIR domain ‘Outer setting’). Providers who recognise a specific need for a programme will also be more likely to implement it with higher fidelity (Durlak & DuPre, 2008). The decision as to whether or not to introduce a programme is strongly influenced by whether the staff believe it can actually be integrated into school routines, as they do not want to put effort and time into something that cannot be sustained (CFIR domain ‘Intervention Characteristics’). The HSS programme is designed for pre-school classes of 5–7 years old children but, as the nurses and school principals in our study pointed out, the attitudes and ideas can be supported by all school professionals during all grades. Teachers, school nurses and meal staff could contribute to implementing and sustaining such a whole-school programme by acting as role models, initiating discussions and offering healthy alternatives.

Participants in this study had to come to terms with the circumstance that in a future implementation study MI would have to be carried out by the school nurses themselves, and not by an outside expert as in our previous studies (CFIR domain ‘Intervention Characteristics’). As our study’s school nurses already perceived their workload as overburdened, their main focus was on feasibility. Therefore, using MI as a component may require additional resources in terms of time, money and training.

MI has been found effective in promoting healthy diet and physical activity behaviours in adults (Hardcastle, Taylor, Bailey, Harley, & Hagger, 2013; Martins & McNeil, 2009). The technique has previously been used in programmes that involve parental support (Dawson et al., 2014; Schwartz et al., 2007) and it is often appreciated by parents. Being client-centred, MI is a flexible method that can be adapted to the severity of the concern, degree of motivation, and wishes of the specific parent. A Danish study concerning the use of MI in school health services revealed that the school nurses perceived it as useful in working with both parents and children to prevent overweight and obesity (Bonde, Bentsen, & Hindhede, 2014). The majority of school nurses in our study had training and some experience in MI already. However, previous studies show that in general, the MI conducted within health care services does not meet recommended standards for MI competence (Ostlund, Kristofferzon, Haggstrom, & Wadensten, 2015). MI is generally learnt over time and both practice and supervision are essential (Miller, Yahne, Moyers, Martinez, & Pirritano, 2004). Hence, although many school nurses in Sweden already have some basic training in MI, additional practice with supervision and feedback on audio-recorded MI sessions would be needed if they are to attain full MI competence. This, in turn, is costly, but as MI appears effective in health promotion, such training could be cost-effective.

One argument against introduction of a programme like HSS was uncertainty about whether it is the role of the school to provide support to parents, and school nurses asked for policy guidance in this regard (CFIR domain ‘Outer setting’). The same uncertainty about the boundary between parents’ and schools’ responsibility when it comes to healthy eating and sufficient physical activity has previously been noted in the international literature (Clarke, Fletcher, Lancashire, Pallan, & Adab, 2013) and might lead to lack of appropriate action. As a matter of fact, the Swedish guideline for school health care from 2014, and updated in 2016, does encourage schools to support parents (The National Board of Health and Welfare & The Swedish National Agency for Education, 2016). But because of the high workload there is still uncertainty about whether or not it is right to focus on supporting parents. A policy decision on health promotion and parental support, preferably including additional funding, could provide a further incentive for schools when they must prioritise actions. This finding is in line with a study by Clarke et al. (2017), who interviewed head teachers regarding obesity prevention in English primary schools. Like our respondents, school leaders expressed a need for support through resources and government policy in order to fulfil this role. It is our impression that the school food environment in Sweden is conducive to health due to policies at the local and national levels to improve school meal quality (Patterson & Elinder, 2015) and remove unhealthy food products from primary schools. In addition, the government decided to add extra hours of physical education to the curriculum in primary schools as of 2019. Furthermore, the Swedish government has made additional funding available for student health care staff since 2016, with no prioritisation regarding the focus area.

Another interesting result of the study concerns the issue of whether a top*–*down decision to conduct a health programme would help or impede implementation (‘Implementation process’ in CFIR). On the one hand, study participants reported that a top*–*down decision may disengage staff. Earlier research shows that school staff pressured to offer new programmes do not implement them very effectively, probably because they are not committed enough (Durlak & DuPre, 2008). Our previous implementation study of HSS also supports this observation. Teachers who were told to carry out the programme felt they were being forced, which affected their engagement in the programme negatively (Norman, Nyberg, Elinder, & Berlin, 2016). To avoid opposition, staff should be actively involved in detailed planning regarding when and how the programme should be implemented. According to a systematic review on implementation of the concept of health-promoting schools, enthusiasm among staff is maintained if they have a sense of ownership, which can be achieved by letting them play an important role in strategic planning and decision-making (Hung et al., 2014).

It became clear that to create commitment among staff and implement the obesity prevention intervention with high fidelity, the staff should be offered training and opportunities to discuss the content of the programme. Our second trial showed that teachers’ time for making the necessary preparations for the intervention and doing so before finalising their plans for the school year, influenced their engagement in the programme (Norman et al., 2016). Furthermore, the results of this study demonstrated that lack of parental engagement is a barrier to securing parental support. Both process evaluations from the earlier trials confirm that successful implementation to a large extent relies on good cooperation between home and school. The importance of facilitating communication and clearly defining the division of responsibilities between project management (i.e., researchers), schools, and parents is emphasized (Bergstrom et al., 2015). It is also important to tailor the intervention to the abilities of the target group to increase participant engagement (Norman et al., 2016).

To support implementation, participants desired a kick-off meeting with an inspirational person from outside the organisation. This kind of ‘external change agent” is described in CFIR as individuals affiliated with an outside entity, who influence or facilitate the implementation (Damschroder et al., 2009). Such a kick-off meeting could function to engage parents as well as school staff and contribute to enhancing cooperation between school and parents regarding the programme.

### Strengths and Weaknesses

Prior to implementing a programme, capacity and needs assessment must be carried out to identify potential barriers and facilitators from the perspective of the individuals involved in the implementation (Damschroder et al., 2009). However, the participants in this study had no prior experience of the programme, except reading the manual 1 week before the interview. On the other hand, they had knowledge and experience from the setting where the programme is to be implemented, which we considered important. We collected data from three different groups of professionals, which increases the trustworthiness of the study (Patton, 2015). Trustworthiness was also increased by illustrative quotes and intersubjective agreement in the coding and analysis of the data (Patton, 2015).

We asked each participating municipality to aim at including participants from areas representing variations in SES. However, a purposive sampling of schools with maximum variation (Patton, 2015) with regard to area SES would have been more appropriate to make sure that full range of this characteristic was represented. This was not possible because of a restricted time frame and difficulties recruiting enough informants, due to the fully booked schedules of school nurses and school principals. Another weakness was the limited number of participants in one of the focus groups.

As the setting is described in detail, and the results are referred back to the guiding framework (CFIR), the results of this study should provide useful guidance for implementation of similar health promotion interventions in the school context.

## Conclusions

When implementing a parental support programme to promote physical activity and healthy dietary habits for children within a school context, it is crucial to create commitment among all staff. The resources available to schools are scarce, and in order for staff members to prioritise such a programme, it should be based on needs, have policy support, be integrated into routine school practice, and seek to improve both health and learning for the children. Barriers to implementation included financial and time constraints, other health needs competing for resources, and challenges in engaging parents. To summarise, the implementation of a parental support programme in school can be facilitated by factors external and internal to the organisation, and intervention characteristics. The external factors comprise support from decision-makers through policies, guidelines and financial incentives as well as access to external support by phone or email, and expert guidance through an inspirational kick-off meeting. Important intervention characteristics were found to facilitate implementation such as a clearly structured manual including detailed information and checklists, and information materials to use when presenting the programme to parents.

Internal factors facilitating implementation include use of pre-existing resources, such as competent and engaged staff, multidisciplinary health care teams, web-based systems for documentation and communication, municipality networks, and local experts. Other important internal factors for effective implementation include the integration of the programme into the school routines and creating awareness among all staff as well as appointment of a multidisciplinary team and an implementation leader at each school, to carry out detailed planning and time management.
